# Pseudorapidity dependence of the bulk properties of hadronic medium in *pp* collisions at 7 TeV

**DOI:** 10.1038/s41598-022-11685-9

**Published:** 2022-05-17

**Authors:** Muhammad Ajaz, Abd Al Karim Haj Ismail, Muhammad Waqas, Mais Suleymanov, Atef AbdelKader, Rustam Suleymanov

**Affiliations:** 1 Department of physics, Abdul Wali Khan University Mardan, Mardan, 23200 Pakistan; 2 College of Humanities and Sciences, Ajman University, Ajman, 346 United Arab Emirates; 3 Nonlinear Dynamic Research Center (NDRC), Ajman University, Ajman, 346 United Arab Emirates; 4 School of Nuclear Science and Technology, University of Chinese Academy of Sciences, Beijing, 100049 China; 5 Baku State University, Baku, Azerbaijan; 6 Institute of Physics, National Academy of Sciences, Baku, Azerbaijan

**Keywords:** Physics, Nuclear physics, Particle physics

## Abstract

The measured charged particle $p_T$ spectra in proton-proton collisions obtained by the CMS experiment at CERN is compared with the simulation results of EPOS–LHC and Pythia8.24 models at 7 TeV center-of-mass energy. The Pythia8.24 model describes the experimental data very well, particularly in the high $p_T$ region. The model also predicts the $p_T$ spectra for $|$ $\eta$ $|$ < 2.4 at 0 $\le$ $p_T$ $\le$ 6 $\text {GeV/}c$. The EPOS–LHC model underpredicts the $p_T$ spectra from 0.1 to 2 $\text {GeV/}c$ in all $\eta$ bins for about 20% and the $p_T$ spectrum from 0.1 to 4.2 $\text {GeV/}c$ for $|$ $\eta$ $|$ < 2.4 by about 15% while reasonably predicts well for $p_T$ > 4.2 $\text {GeV/}c$ within the experimental errors. Furthermore, to get information about collective properties of the hadronic matter, modified Hagedorn function with embedded transverse flow velocity and thermodynamically consistent Tsallis distribution functions are used to fit the experimental data and simulated results. The values of $\chi ^2/ndf$ show that the functions fit the data and simulation results well. The parameter extracted by the functions: $\beta _T$, $T_0$, and $T_{eff}$ decreases with increasing $\eta$. The decrease in $\beta _T$ with increasing $\eta$ is due to the large energy deposition in lower rapidity bins producing rapid expansion due to large pressure gradient resulting quick expansion of the fireball. Similarly, large energy transfer in the lower pseudo-rapidity bin results in higher degree of excitation of the system which results larger values of $T_0$ and $T_{eff}$. The values of the fit constant $N_0$ increase with $\eta$ where the values of $N_0$ extracted from Pythia8.24 are closer to the data than the EPOS–LHC model. The Pythia8.24 model has better prediction than the EPOS–LHC model which might be connected to its flow-like features and color re-connections resulting from different Parton interactions in the initial and final state.

## Introduction

High energy particle collision is a very complex topic that makes it hard to measure and understand the geometry, quantities, and global properties of the collisions. Therefore, in addition to the measurements of some quantities by experiments, one must rely on theoretical models to explain some other characteristics based on experimental results. Experiments using high energy *pp* collisions such as the Relativistic Heavy Ion Collider (RHIC)^1,2^ and the Large Hadron Collider (LHC) have been very useful to study the characteristics of these collisions such as the flow effects, the pseudorapidity, and the transverse momentum distributions. On the other hand, different models have been presented in the literature which tried to describe the collision process at different stages^3,4^.

A measurement of pseudorapidity density provides constraints to the modeling of the characteristics of *pp* collisions such as the direction of particle emission. The pseudorapidity ($\eta$) is given by $\eta = - \ln (\tan (\theta /2))$, where $\theta$ is the polar angle that the charged particles make with the anticlockwise beam direction. The presented analysis are of $dN_{ch}$ / d$\eta$ and $dN_{ch}/dp_T$ in a pseudorapidity range of $|$ $\eta$ $|$ < 2.4 in steps of 0.2. In the current analysis, the charged hadrons ($N_{ch}$) includes decay products of particles with a lifetime of < 1 cm, while the products of secondary particles are excluded and a correction factor is applied for prompt leptons.

The $p_T$ and $\eta$ distributions of charged particles, pions, kaons, protons, and anti-protons produced in nucleus-nucleus, hadron-nucleus, and *pp* collisions are very important observables because they provide very crucial information about the anisotropy and dynamics of the final state particles produced in collisions at high energies. In addition, $p_T$ spectra are different for different particles in the range of 0 to 100 $\text {GeV/}c$, which makes it difficult to explain how wide is the distribution range for different energies. However, one can discuss different $p_T$ ranges including very low, low, intermediate, high, and very high regions^5–10^. Generally, $p_T$ spectra are the combination of two main processes, soft and hard processes. The soft processes are dominant at the low $p_T$ range and the hard processes become more pronounced at higher $p_T$ ranges. Furthermore, similar results are reported from predictions of different model simulations of the $p_T$ distributions at different energies^11–14^.

In this work, we contrasted the simulation results of the transverse momentum ($p_T$) distributions of the charged particles with the measurements of the experimental data in *pp* collisions at 7 TeV that are further analyzed by statistical functions to extract the bulk properties of the hadronic matter. The temperature at the kinetic freeze-out stage ($T_0$), the effective temperature ($T_{eff}$) and the average transverse flow velocity ($\beta _T$) are extracted by fitting the data with these statistical functions. $T_0$ and $<\beta _T>$ are obtained by using a modified Hagedorn function with embedded transverse flow velocity^15–18^. In addition, $T_{eff}$ is extracted by using the Tsallis distribution function^19–23^. The experimental data are compared with the Monte Carlo models predictions elaborated in the following “Method and models” section. Fits explained in “Method and models” section is applied to the models’ simulations as well as experimental data for better comparisons. It is worth mentioning that the error bars in the experimental data are the quadrature sum of systematic and statistical errors while in the case of models simulations only the statistical errors are shown. No error bars are used where fit curves are shown on data and model simulations.

The rest of this article is organized as follows: “Method and models” section presents an overview of the methods and Monte Carlo models’ simulations under study. In “Results and discussion” section, the details of the analysis procedure, and the results of the simulations using EPOS^24^ and Pythia^25^ along with a comparison to experimental CMS data^26^ and the fitting with theoretical functions, are presented. Finally, the summary and conclusions are given in “Summary and conclusion” section.

## Method and models

Pseudorapidity $(\eta$) distributions in *pp* collisions at 7 TeV are obtained from the inner tracking system of the Compact Muon Solenoid experiment (CMS) at the Large Hadron Collider (LHC)^26^ on March 30, 2010. The spectra obtained by the experiment were normalized to all non single diffractive events with corrections for trigger and selection efficiency, acceptance, and branching ratios. Simulations of two Monte Carlo models, EPOS^24^ and Pythia^25^ at the same energy are performed and are then compared with the measurements from the experimental data. These models are based on the simple Parton model using theory of Reggie-Gribov^27^, which is a QCD based effective field theory that accounts for multiple interactions in parallel with Pomerons considered to represent the partons interactions^28^. A brief description about each of these models is provided below.

The generator EPOS^24^ is a hadronic interaction Monte Carlo simulation package that is well known to simulate the hadronic interactions in high energy cosmic ray simulations. In addition, it is used to describe the minimum bias interactions and centrality dependence of heavy-ion collisions. EPOS is an acronym of Energy conserving quantum mechanical multiple scattering approach based on Partons Off-shell remnants and Splitting of parton ladders. Soft, semi-soft and hard Pomeron exchanges are used to describe the interaction between particles in EPOS, where the particle production originates from two kind of sources, cut Pomeron and remnant decays^29^. In its last improvement, EPOS was updated to a new version, called EPOS-LHC^24^, to cover the energies of the LHC. The new version can reproduce all minimum bias results of all particles with transverse momentum from 0 to a few $\text {GeV/}c$. In addition, in case of very dense system in a small volume in *pp* collisions, a different parametrization of flow has been introduced in EPOS, compared to the large volume produced in heavy-ion collisions. In this paper, we have used the EPOS-LHC version of the EPOS model but for simplicity only EPOS will be used throughout the manuscript.

Pythia is one of the most widely used Monte Carlo event generator of particle collisions in high energy physics with emphasis on *pp* interactions^25^. The hadronization process of transforming the final outgoing coloured Partons into colourless particles is based on the Lund string fragmentation model^30^. The main event in a *pp* collision can be represented by a large number of processes, such as elastic and diffractive processes, electroweak processes, QCD hard and soft processes and top quark production. The model also implements the initial and final state radiations, multi Partonic interactions, beam remnants which are formed after the extraction of multi Partonic interactions^31^. We used Phythia8.24 version of the Pythia model but for simplicity we will be using Pythia throughout the manuscript. It is also pertinent to mention that one million events are simulated in case of both the models.

In a standard analysis, it is always important to compare the results of the simulation generated with the Monte Carlo models to experimental data. In addition to the comparison of models’ prediction with the experimental data, we use the theoretical Hagedorn function^32^ to fit the experimental data as well as the models’ simulations to extract the freeze-out parameters. The Hagedorn function, a QCD -inspired inverse power law, which can produce the transverse mass $m_T$ distribution of hadrons in *pp* and *AA* collisions. This Hagedorn function describes the bulk spectra in the low transverse mass region as well as the particles produced in QCD hard scatterings. The function is given as:

1 $$\begin{aligned} \frac{d^2N}{2\pi N_{ev}p_{T}dp_{T}dy} = C\bigg (1+\frac{m_T}{p_0}\bigg )^{-n}. \end{aligned}$$

Here, $m_{t} = \sqrt{p_T^2 + m_0^2}$ is the transverse mass of hadrons, $m_0$ the rest mass while *C* is the normalization constant. The *n* and $p_{0}$ in the equation are two free parameters of the function. Moreover, the Tsallis function^15–18^ can excellently describe the $p_T$ and $m_T$ invariant distribution measured in *pp* collisions at high energies. There are several version of the Tsallis function that can give good fit results to the $p_T$ spectra, however, the following expression is a simple version of the Tsallis function^22^ that describes the invariant spectra of particles in terms of the effective temperature $T_{eff}$ and non-extensivity parameter *q* which accounts for the deviation of the $p_T$ spectra from the usual Boltzmann–Gibbs exponential distribution function. This version of the Tsallis function is a consistent version of thermodynamics for the particle number, energy, density and pressure at mid-rapidity ($y \approx 0$), and is given by the following expression:

2 $$\begin{aligned} \frac{d^2N}{2\pi N_{ev}p_{T}dp_{T}dy} = C_q\bigg (1+(q-1)\frac{m_T}{T_{eff}}\bigg )^{-1/(q-1)}. \end{aligned}$$

where $C_q$ is constant of the fit function, $T_{eff}$ is the effective temperature, *q* is the non-extensive parameter and can also be considered as a measure of the non-thermalization^23^. When the parameter *q* is close to one, the thermalization degree of a system is larger and the Tsallis distribution approaches the normal Boltzmann–Gibbs exponential distribution function. On the other hand, the effective temperature parameter $T_{eff}$ represents the contribution from the thermal motion of particles as well as the collective flow of expanding matter^22^.

3 $$\begin{aligned} \frac{d^2N}{2\pi N_{ev}p_{T}dp_{T}dy} = C_qm_T\bigg (1+(q-1)\frac{m_T}{T_{eff}}\bigg )^{-q/(q-1)}. \end{aligned}$$

which is called the thermodynamically consistent Tsallis function throughout the paper or Tsallis function for shortness.

Assuming that $n = (q-1)^{-1}$ and $p_0 = nT$, we notice that Eqs. (1) and (2) are mathematically equivalent. One can see that the parameters *n* and *q* are inversely proportional to each other, as the value of the parameter *n* increases, the parameter *q* decreases.

4 $$\begin{aligned} \frac{d^2N}{2\pi N_{ev}p_{T}dp_{T}dy} = C\bigg (1+\frac{m_T}{nT_0}\bigg )^{-n}. \end{aligned}$$

Finally to include the transverse flow in Eq. (4), $m_{T}$ is equated with

5 $$\begin{aligned} m_{T} =<\gamma _{T}>\bigg (m_{T}-p_{T}<\beta _{T}>\bigg ). \end{aligned}$$

These modifications have been discussed in^18,33,34^ and have successfully calculated the average transverse flow velocity $<\beta _{T}>$ and $T_0$, and the equation is now known as the Hagedorn equation with embedded transverse flow velocity^35^:

6 $$\begin{aligned} \frac{d^2N}{2\pi N_{ev}p_{T}dp_{T}dy} =C \bigg (1+<\gamma _{T}>\frac{m_{T}-p_{T}<\beta _{T}>}{nT_0}\bigg )^{-n}. \end{aligned}$$

where *C* is the normalization constant and is to be normalized to 1. $<\gamma _{t}>$ = $1/\sqrt{1-<\beta _{t}^{2}>}$ and $<\beta _t>$ is the average transverse flow velocity. This simple form of the equation having a few parameters, is very powerful tool to compare different collisions with a small number of parameters. The Hagedorn function has reproduced the spectra described in^18,32,33^ with physical parameters. We can re-arrange these terms in our current analysis as follows,

7 $$\begin{aligned}&\frac{d^2N}{N_{ev}dp_{T}dy} = 2\pi p_{T} C \bigg (1+<\gamma _{T}>\frac{m_{T}-p_{T}<\beta _{T}>}{nT_0}\bigg )^{-n}. \end{aligned}$$

8 $$\begin{aligned}&\frac{d^2N}{N_{ev}dp_{T}dy} = 2\pi C_qp_Tm_T\bigg (1+(q-1)\frac{m_T}{T_{eff}}\bigg )^{-q/(q-1)}. \end{aligned}$$

The $p_{T}$ spectra of charged hadrons from experiment as well as model simulations are fitted with Hagedorn equations (Eq. 7) and thermodynamically consistent Tsallis function (Eq. 8) to extract the fit parameters as explained above. We have used the method of least square for the extraction of the related parameters. The Tsallis function is very effective in providing an excellent description of the transverse momentum spectra of particles, for nucleus-nucleus (AA) as well as hadron-hadron (hh) collisions at high energies. The function has three free parameters including normalization/fitting constant indicating volume information, the non-extensivity parameter used to see the deviation of the distribution from the exponential Boltzman and Gibbs distribution, and the effective temperature which includes the flow information along with the thermal motion of the particles. Furthermore, to describe $p_T$ distribution of particles in AA and hh collisions, different models with flow definitions are included in the Tsallis distribution function. Hagedorn function is one of them which among other parameters gives direct access to the transverse expansion (flow velocity) and kinetic freeze-out temperature with an excellent description of the $p_T$ spectra at high energies. In addition, the modified Hagedorn function is very close to the ideal gas model. Both the Hagedorn and Tsallis models have the advantage that they cover a wide range of $p_T$ due to the entropy parameter *q*(*n*) where $q=1/(n-1)$.

## Results and discussion

The transverse momentum ($p_T$) spectra of charged hadrons simulated with the EPOS and Pythia models are compared with the *pp* collisions at $\sqrt{s} =$ 7 TeV measured by the CMS experiment^26^. The initial conditions used for simulations are similar to that of experimental conditions. The $p_T$ spectra from 0.1 to 2 GeV/*c* in different $\eta$ bins from 0 to 2.4 in steps of 0.2, and the $p_T$ spectrum from 0.1 to 6 GeV for 0 $\le$ $|$ $\eta$ $|$ < 2.4 as a single $\eta$ bin are studied using the aforementioned models.

**Figure 1:**
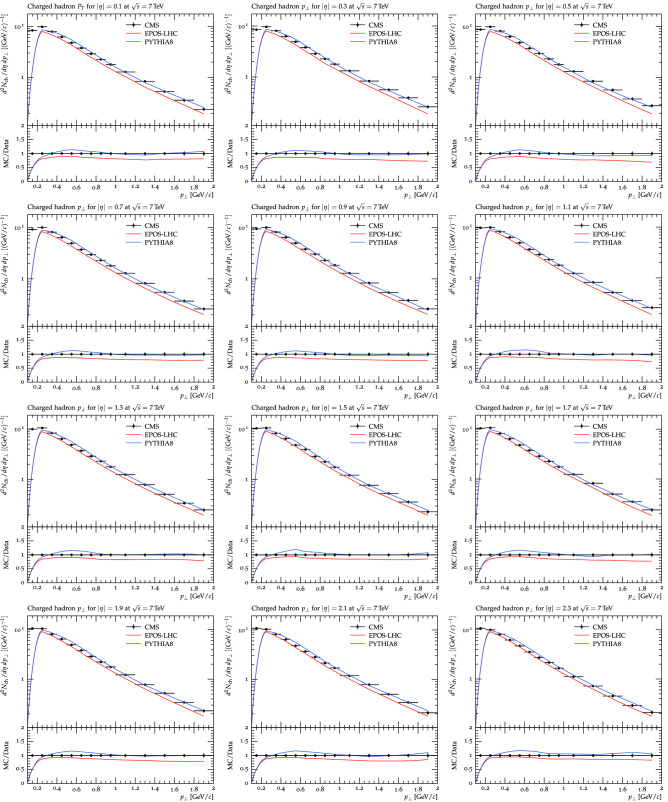
The $p_T$ spectra of all charged hadrons are presented at different pseudorapidity bins measured by the CMS experiment in comparison with the prediction of EPOS and Pythia models. Black solid markers represent the experimental measurements while lines of different colors show the models’ predictions. Blue line shows the Pythia while the red line is used for the EPOS model’s prediction.

Figure 1 shows the $p_T$ distributions of charged hadrons in different $\eta$ bins starting from 0.1 (corresponding to $\eta = 0.0{-}0.2$) from top left to $\eta = 2.3$ ($2.2{-}2.4$) to the bottom right. The experimental data is shown by solid black markers while lines of different colors represent the two models calculations. A red solid line is used to show the results of the EPOS model, while the blue solid line is for Pythia results. The quadrature sum of the systematic and statistical errors are considered in the experimental results, while model calculations include the statistical errors only. The horizontal error bars show the bin size shown in the experimental results. The two event generators have the same horizontal bin size in $p_T$ as the experimental data. It has been observed that for $p_T$ above 0.8 $\text {GeV/}c$, Pythia measurements fully describe the $p_T$ spectra at all the pseudorapidity regions. Below 0.3 $\text {GeV/}c$, Pythia model under-predicts the experimental data, while for 0.3 $\le$ $p_T$ $\le$ 0.8 $\text {GeV/}c$, the model slightly overshoot the experimental data which is about 10%. There is no significant $\eta$ dependence observed in the model’s predictions and have similar results for all pseudorapidity regions. The EPOS model under-predicts the experimental data over the entire $p_T$ range up to about 20% and is also observed to be independent of all $\eta$ regions understudy. The data to Monte Carlo ratio is shown in the lower panel of each plot which supports the above statement.

**Figure 2:**
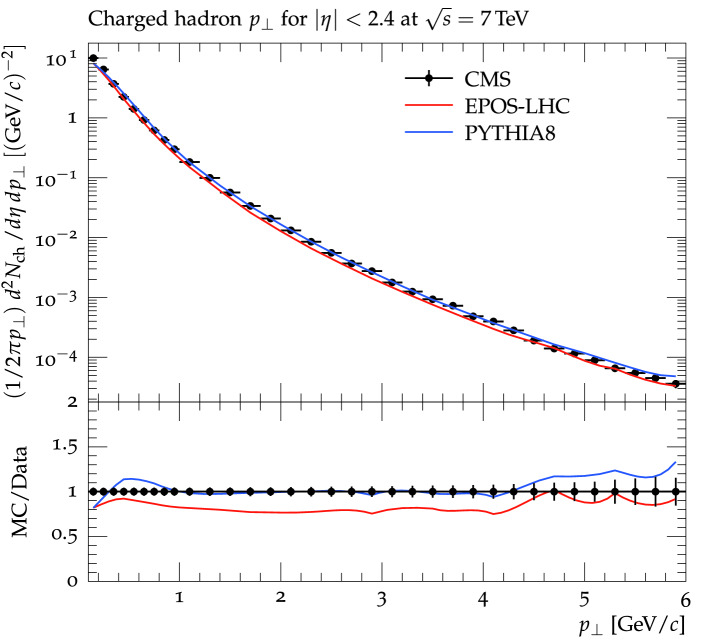
The $p_T$ spectrum of charged hadrons at $|$ $\eta$ $|$ < 2.4 measured by the CMS experiment compared with the prediction of the EPOS and Pythia models. Solid black markers represents the experimental measurements while lines of different colors show the models’ predictions. A blue line shows the Pythia prediction while the red line represents the EPOS model predictions.

Figure 2 shows the predictions of the $p_T$ spectra of the two models in comparison to the experimental data for $|$ $\eta$ $|$ < 2.4 $\text {GeV/}c$. Again the Pythia model reproduced the experimental data over most of the $p_T$ region within the experimental errors. For $p_T$ < 1 $\text {GeV/}c$, the model slightly overshoot the data in a narrow region of the $p_T$ for about 10%. The EPOS model again underestimates the experimental data over the entire $p_T$ region with decreasing discrepancy with increasing $p_T$. The ratio of the Monte Carlo predictions to the data is also shown in the lower panel of the plot which supports our statements.

**Figure 3:**
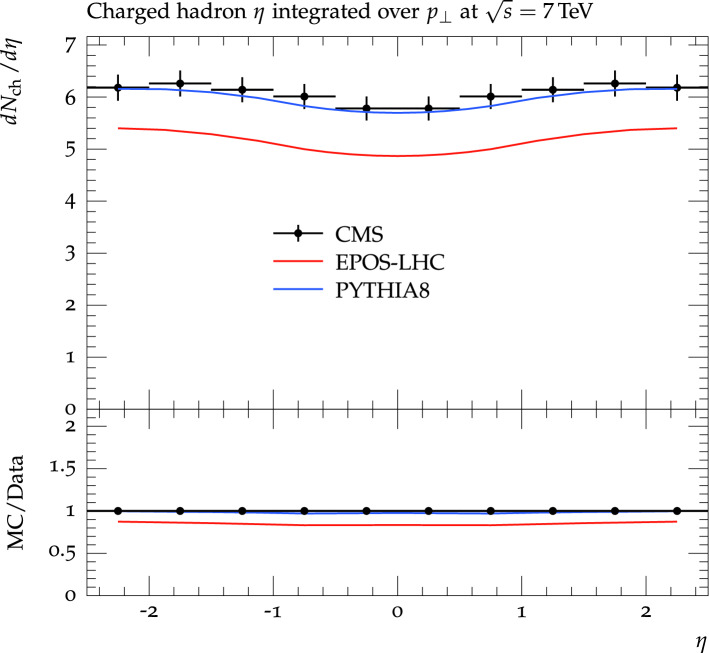
The charged hadrons $\eta$ integrated over $p_T$ at − 2.5 $\le$ $\eta$ $\le$ 2.5 measured by the CMS experiment is compared with the predictions of the EPOS and Pythia models. Solid black markers represent the experimental measurements while lines of different colors show the models’ predictions. A red line shows the Pythia’s predictions while the blue line represents the EPOS model predictions.

Figure 3 shows the $\eta$ of charged hadrons integrated over the $p_T$ for − 2.5 $\le$ $\eta$ $\le$ 2.5 in comparison with the models’ predictions. Pythia model reproduced the experimental results of $dN_{ch}/d\eta$ very well for the whole $\eta$ region, − 2.5 $\le$ $\eta$ $\le$ 2.5. The EPOS model does not reproduce the data and hence underpredicts the charged particles $\eta$ integrated over $p_T$ over the entire region of − 2.5 $\le$ $\eta$ $\le$ 2.5 from 10% to about 15%. The lower panel of the graph shows the ratio of the Monte Carlo predictions to the data. The description mentioned can easily be inferred from the ratio plot as well.

**Figure 4:**
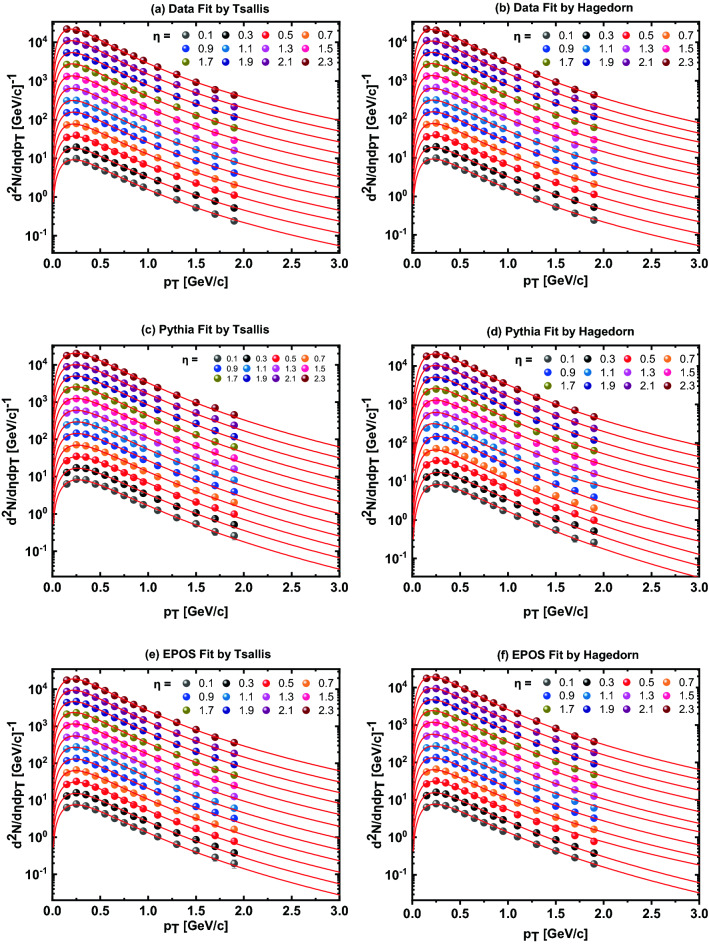
The charged hadrons $p_T$ spectra in different $\eta$ intervals from 0 to 2.4 in steps of 0.2 measured by the CMS experiment is fitted with the Tsallis function (Eq. 7) (left) and Hagedorn function (Eq. 8) (right) shown in the first row (**a** and **b**). Same markers with different colors are used to represent each slice of $\eta$ bin. Lines of the same color are used to show the fit results on the experimental data. The second (**c** and **d**) and third (**e** and **f**) rows show the Pythia and EPOS simulations fitted with the Tsallis (left) and Hagedorn (right) functions, respectively.

To extract the bulk properties of the hadronic matter created as a result of *pp* collisions at $\sqrt{s} =$ 7 TeV, the CMS experimental data^36^ and models’ simulations are fitted with two statistical functions. The fit results of the data and two models’ predictions are presented in Fig. 4 which show that the fit functions fit the data very well. The experimental data and model simulations are fitted by the modified Hagedorn function with embedded transverse flow velocity (Eq. 7) and thermodynamically consistent Tsallis distribution function (Eq. 8). Figure 4a,b show the $p_T$ spectra of the experimental data in different $\eta$ intervals fitted with the modified Tsallis and Hagedorn functions respectively. Similarly, Fig. 4c,d show the fit results of the two function on Pythia model’s prediction while Fig. 4e,f show the fit results of the two statistical models on the EPOS model’s predictions.

The spectra at different $\eta$ intervals from 0 to 2.4 in steps of 0.2 respectively are scaled by 1, 2, 4, 8, 16, 32, 64, 128, 254, 512, 1024, and 2048 respectively for better visualisation. The values of the extracted parameters obtained from the fit by Hagedorn distribution function using Eq. (7) are listed in Table 1. *The values of* $\chi ^2/ndf$ also show that the two functions fit the data and models’ predictions very well given in the last column of the table. The values of transverse flow velocity ($\beta _T$), *n*, and Kinetic freeze-out temperature ($T_0$) are extracted by fitting the spectra of the experimental data and models’ simulations tabulated as a third, fourth, and fifth column in the Table 1. These parameters are directly connected to the scattering centers involved in the interaction process.

**Table 1:** The values of free parameters ($T_0$ and $\beta _T$), normalization constant ($N_0$), *n*, and $\chi ^2/ndf$ at different $\eta$ values from 0 to 2.4 in steps of 0.2 using modified Hagedorn function with embedded transverse flow velocity using Eq. (7) for the CMS data, Pythia model, and EPOS model.

Model	$\eta$	$N_0$	$\beta _T (GeV/c)$	*n*	$T_0 (MeV)$	$\chi ^2/ndf$
CMS data	0.0–0.2	$92.8\pm 0.1$	$0.3836\pm 0.0002$	$5.749\pm 0.001$	$76.37\pm 0.01$	3.9416/11
	0.2–0.4	$94.0\pm 0.1$	$0.3979\pm 0.0002$	$5.749\pm 0.001$	$75.85\pm 0.01$	2.8339/11
	0.4–0.6	$95.0\pm 0.1$	$0.3990\pm 0.0002$	$5.738\pm 0.001$	$75.45\pm 0.01$	4.3806/11
	0.6–0.8	$96.0\pm 0.1$	$0.3767\pm 0.0002$	$5.738\pm 0.001$	$76.12\pm 0.01$	2.6043/11
	0.8–1.0	$96.8\pm 0.2$	$0.3765\pm 0.0002$	$5.736\pm 0.001$	$76.18\pm 0.01$	2.7891/11
	1.0–1.2	$96.4\pm 0.2$	$0.3602\pm 0.0002$	$5.702\pm 0.001$	$76.23\pm 0.01$	3.9972/11
	1.2–1.4	$97.5\pm 0.1$	$0.3420\pm 0.0002$	$5.545\pm 0.001$	$76.25\pm 0.01$	1.9827/11
	1.4–1.6	$98.1\pm 0.1$	$0.3281\pm 0.0002$	$5.690\pm 0.002$	$76.28\pm 0.01$	5.0891/11
	1.6–1.8	$99.5\pm 0.1$	$0.3260\pm 0.0002$	$5.538\pm 0.002$	$73.45\pm 0.01$	5.5269/11
	1.8–2.0	$99.8\pm 0.1$	$0.3220\pm 0.0002$	$5.538\pm 0.002$	$73.25\pm 0.01$	4.3067/11
	2.0–2.2	$98.0\pm 0.2$	$0.3490\pm 0.0002$	$5.875\pm 0.012$	$74.66\pm 0.01$	9.4020/11
	2.2–2.4	$96.0\pm 0.1$	$0.3505\pm 0.0002$	$5.84\pm 0.012$	$71.86\pm 0.01$	6.8266/11
Pythia model	0.0–0.2	$88.0\pm 0.1$	$0.6178\pm 0.0002$	$7.529\pm 0.002$	$67.7\pm 0.02$	3.3603/11
	0.2–0.4	$90.0\pm 0.1$	$0.6166\pm 0.0002$	$7.510\pm 0.002$	$67.5\pm 0.02$	3.3568/11
	0.4–0.6	$90.5\pm 0.1$	$0.6116\pm 0.0002$	$7.507\pm 0.002$	$67.5\pm 0.02$	2.3721/11
	0.6–0.8	$80.5\pm 0.1$	$0.5876\pm 0.0002$	$6.057\pm 0.002$	$54.5\pm 0.02$	1.6427/11
	0.8–1.0	$93.0\pm 0.1$	$0.6704\pm 0.0002$	$6.808\pm 0.002$	$51.8\pm 0.02$	1.4146/11
	1.0–1.2	$95.0\pm 0.1$	$0.510\pm 0.0022$	$5.101\pm 0.121$	$51.8\pm 0.02$	5.1238/11
	1.2–1.4	$96.5\pm 0.1$	$0.610\pm 0.0002$	$5.912\pm 0.002$	$51.9\pm 0.02$	1.3226/11
	1.4–1.6	$97.0\pm 0.1$	$0.604\pm 0.0002$	$5.912\pm 0.001$	$52.1\pm 0.01$	1.7307/11
	1.6–1.8	$97.5\pm 0.1$	$0.601\pm 0.0002$	$5.945\pm 0.001$	$52.0\pm 0.01$	1.1803/11
	1.8–2.0	$97.5\pm 0.1$	$0.600\pm 0.0002$	$5.945\pm 0.001$	$51.7\pm 0.02$	1.3284/11
	2.0–2.2	$97.0\pm 0.1$	$0.598\pm 0.0002$	$5.945\pm 0.001$	$51.7\pm 0.01$	1.8547/11
	2.2–2.4	$96.5\pm 0.1$	$0.593\pm 0.0002$	$5.945\pm 0.001$	$51.7\pm 0.02$	1.2900/11
EPOS-LHC model	0.0–0.2	$78.0\pm 0.1$	$0.482\pm 0.0002$	$6.678\pm 0.001$	$76.0\pm 0.02$	2.4992/11
	0.2–0.4	$77.5\pm 0.1$	$0.467\pm 0.0002$	$6.678\pm 0.001$	$75.9\pm 0.01$	1.8971/11
	0.4–0.6	$95.0\pm 0.2$	$0.417\pm 0.0002$	$5.77\pm 0.001$	$74.5\pm 0.02$	5.1725/11
	0.6–0.8	$79.0\pm 0.2$	$0.415\pm 0.0002$	$5.98\pm 0.002$	$74.5\pm 0.02$	2.3327/11
	0.8–1.0	$81.5\pm 0.1$	$0.406\pm 0.0002$	$5.98\pm 0.002$	$74.5\pm 0.02$	1.2255/11
	1.0–1.2	$96.0\pm 0.1$	$0.389\pm 0.0002$	$5.79\pm 0.001$	$74.5\pm 0.02$	5.7511/11
	1.2–1.4	$84.0\pm 0.1$	$0.381\pm 0.0002$	$5.99\pm 0.001$	$74.5\pm 0.02$	0.8810/11
	1.4–1.6	$85.0\pm 0.1$	$0.377\pm 0.0002$	$5.99\pm 0.001$	$74.5\pm 0.01$	0.7250/11
	1.6–1.8	$86.0\pm 0.1$	$0.369\pm 0.0002$	$5.99\pm 0.001$	$74.5\pm 0.02$	0.6412/11
	1.8–2.0	$86.0\pm 0.1$	$0.362\pm 0.0002$	$5.99\pm 0.001$	$74.5\pm 0.01$	0.2543/11
	2.0–2.2	$86.5\pm 0.1$	$0.353\pm 0.0002$	$5.996\pm 0.001$	$74.5\pm 0.01$	0.6915/11
	2.2–2.4	$86.0\pm 0.1$	$0.347\pm 0.0002$	$6.081\pm 0.002$	$74.5\pm 0.01$	0.6222/11

**Table 2:** The values of free parameters (*q* and $T_{eff}$), normalization constant ($N_0$), and $\chi ^2/ndf$ at different values of $\eta$ from 0 to 2.4 in steps of 0.2 using thermodynamically consistent Tsallis distribution function given by Eq. (8) are shown here for the experimental data, Pythia model, and EPOS model.

Model	$\eta$	$N_0$	*q*	$T_{eff} (MeV)$	$\chi ^2/ndf$
CMS fata	0.0–0.2	$93.0\pm 0.2$	$1.185\pm 0.002$	$81.93\pm 0.02$	4.7095/11
	0.2–0.4	$94.1\pm 0.2$	$1.187\pm 0.002$	$82.09\pm 0.02$	2.9418/11
	0.4–0.6	$95.2\pm 0.2$	$1.187\pm 0.001$	$82.17\pm 0.02$	4.2392/11
	0.6–0.8	$96.1\pm 0.2$	$1.187\pm 0.001$	$80.16\pm 0.02$	2.9714/11
	0.8–1.0	$96.9\pm 0.2$	$1.188\pm 0.001$	$79.17\pm 0.02$	2.9565/11
	1.0–1.2	$96.0\pm 0.3$	$1.189\pm 0.002$	$77.57\pm 0.02$	3.8693/11
	1.2–1.4	$97.2\pm 0.2$	$1.189\pm 0.002$	$75.69\pm 0.02$	4.8091/11
	1.4–1.6	$98.4\pm 0.2$	$1.189\pm 0.002$	$75.45\pm 0.02$	5.8391/11
	1.6–1.8	$99.2\pm 0.2$	$1.189\pm 0.033$	$74.05\pm 0.02$	6.7898/11
	1.8–2.0	$99.3\pm 0.2$	$1.190\pm 0.001$	$73.55\pm 0.02$	5.3094/11
	2.0–2.2	$99.4\pm 0.2$	$1.190\pm 0.003$	$72.06\pm 0.02$	6.7880/11
	2.2–2.4	$97.1\pm 0.2$	$1.192\pm 0.002$	$69.46\pm 0.02$	6.4629/11
Pythia model	0.0–0.2	$88.8\pm 0.2$	$1.140\pm 0.002$	$108.24\pm 0.2$	4.4459/11
	0.2–0.4	$90.4\pm 0.2$	$1.140\pm 0.002$	$107.94\pm 0.02$	4.5186/11
	0.4–0.6	$91.4\pm 0.2$	$1.140\pm 0.002$	$106.84\pm 0.02$	3.3732/11
	0.6–0.8	$92.7\pm 0.2$	$1.140\pm 0.002$	$105.83\pm 0.02$	2.8549/11
	0.8–1.0	$94.1\pm 0.2$	$1.142\pm 0.002$	$103.75\pm 0.02$	2.7462/11
	1.0–1.2	95.7$\pm 0.2$	$1.142\pm 0.002$	$102.73\pm 0.02$	2.5065/11
	1.2–1.4	$97.0\pm 0.2$	$1.146\pm 0.002$	$100.00\pm 0.02$	2.0923/11
	1.4–1.6	$97.5\pm 0.2$	$1.144\pm 0.002$	$100.00\pm 0.02$	2.3008/11
	1.6–1.8	$98.1\pm 0.2$	$1.146\pm 0.002$	$97.70\pm 0.02$	1.6759/11
	1.8–2.0	$98.2\pm 0.2$	$1.147\pm 0.002$	$96.95\pm 0.02$	1.1737/11
	2.0–2.2	$98.2\pm 0.2$	$1.148\pm 0.002$	$95.58\pm 0.02$	1.5751/11
	2.2–2.4	$98.4\pm 0.2$	$1.148\pm 0.002$	$94.75\pm 0.02$	1.4281/11
EPOS-LHC model	0.0–0.2	$77.2\pm 0.2$	$1.150\pm 0.002$	$97.26\pm 0.02$	2.5239/11
	0.2–0.4	$77.4\pm 0.2$	$1.151\pm 0.002$	$96.28\pm 0.02$	2.4604/11
	0.4–0.6	$78.2\pm 0.2$	$1.153\pm 0.002$	$95.04\pm 0.02$	1.8541/11
	0.6–0.8	$79.6\pm 0.2$	$1.154\pm 0.002$	$94.12\pm 0.02$	2.5161/11
	0.8–1.0	$80.8\pm 0.2$	$1.156\pm 0.002$	$91.41\pm 0.02$	2.3710/11
	1.0–1.2	$82.5\pm 0.2$	$1.158\pm 0.002$	$89.46\pm 0.02$	1.5124/11
	1.2–1.4	$83.3\pm 0.2$	$1.161\pm 0.002$	$88.11\pm 0.02$	1.8027/11
	1.4–1.6	$84.8\pm 0.2$	$1.163\pm 0.002$	$86.25\pm 0.02$	1.6193/11
	1.6–1.8	$85.6\pm 0.2$	$1.164\pm 0.002$	$85.15\pm 0.02$	1.0484/11
	1.8–2.0	$86.1\pm 0.2$	$1.165\pm 0.002$	$84.13\pm 0.02$	0.9275/11
	2.0–2.2	$86.2\pm 0.2$	$1.166\pm 0.002$	$82.32\pm 0.02$	0.7033/11
	2.2–2.4	$86.3\pm 0.2$	$1.167\pm 0.002$	$81.38\pm 0.02$	0.4957/11

The values of different parameters are extracted by fitting the experimental data and models’ simulations in each $\eta$ bin for better comparison of predictions with the measurements. The values of $N_0$, $\beta _T$, and $T_0$ decrease with $\eta$ in data and both the models. The values of $\beta _T$ and $T_0$ extracted by the fit functions from the EPOS model are closer to the experimental data than the Pythia model while opposite in the case for $N_0$ where the latter has similar values in Pythia with the data than the EPOS model.

In case of experimental data, the function described in Eq. (7) gives the value of $\beta _T = (0.3836\pm 0.0002$) c for $\eta = 0.1$, which corresponds to the $\eta$ range from 0.0 to 0.2. The extracted value of $\beta _T = (0.3505\pm 0.0002$) c for the $\eta = 2.3$. In the case of Pythia model, the value of $\beta _T$ varies from ($0.6178\pm 0.0002$) c for $\eta = 0.1$ to ($0.593\pm 0.0002$) c for $\eta = 2.3$, whereas it varies from ($0.482\pm 0.0002$) c to ($0.347\pm 0.0002$) c in case of EPOS model for the first and last regions of $\eta$. It has been observed that the variation in the value of $\beta _T$ is monotonic in all the cases. The kinetic freeze–out temperature $T_0$, extracted by fitting the experimental data with Hagedorn function also shows a decreasing trend with increasing $\eta$ starting from ($76.37\pm 0.01$) MeV at $\eta = 0.1$ to ($71.86\pm 0.01$) MeV at $\eta$ = 2.3. The reason behind this decreasing trend of $T_0$ is the decrease of the energy transfer in the system due to the large penetration between participants particles as the system goes from mid-rapidity to the forward-rapidity region and this result is in agreement with our recent work^37^. A similar decreasing trend of the $T_0$ is observed by applying the fit function on the two models’ predictions. In both of the cases, the value of $T_0$ decreases with increasing $\eta$. The highest and lowest values of $T_0$ for Pythia and EPOS models are ($67.7\pm 0.02$) MeV, ($51.7\pm 0.02$) MeV and ($76.0\pm 0.02$) MeV, ($74.5\pm 0.01$) MeV respectively.

The value of *n* obtained from the fitting function has also been observed to show a decreasing trend with increasing $\eta$. The decrease in the values of *n* in case of experimental data is clearer than the data and EPOS model where slight variation in the values of *n* are observed. Furthermore, the values of *n* for EPOS model are closer to the experimental data than the Pythia model which has higher values in all the cases of different $\eta$ intervals. Lastly, the values of normalization constant, $N_0$, shows an increasing trend with increasing $\eta$ intervals in data as well as in both of the models. It increases from $92.8\pm 0.1$ to $96.0\pm 0.1$ in data while from $88.0\pm 0.1$ to $96.5\pm 0.1$ and $78.0\pm 0.1$ to $86.0\pm 0.1$ in Pythia and EPOS models respectively.

The values of different parameter obtained by fitting the experimental data and models’ simulations by thermodynamically consistent Tsallis function are given in Table 2. These parameters include the effective temperature ($T_{eff}$), non-extensivity parameter *q* and normalization constant $N_0$ for different values of $\eta$ from 0 to 2.4 in steps of 0.2. The values of $T_{eff}$ decreases with $\eta$ monotonically in experimental data and in both of the models. It varies from ($81.93\pm 0.02$) MeV to ($69.46\pm 0.02$) MeV in case of data and from ($108.24\pm 0.02$) MeV to ($94.75\pm 0.02$) MeV and ($97.26\pm 0.02$) MeV to ($81.38\pm 0.02$) MeV in cases of Pythia and EPOS models respectively. For a particular $\eta$ bin, both models have higher values of $T_{eff}$ with EPOS has closer value than the Pythia model. The value of *q* has again a monotonically increasing behavior with $\eta$ but now both the models have lower values of *q* than the data. Again the values of *q* in the case of EPOS model has closer values than the Pythia model. The values of *q* varies from $1.185\pm 0.002$ to $1.192\pm 0.002$ in case of experimental data, from $1.140\pm 0.002$ to $1.148\pm 0.002$ in case of Pythia model whereas from $1.150\pm 0.002$ to $1.167\pm 0.002$ in the case of EPOS model.

The relation of *q* with *n* can be established by comparing the two tables resulting from two different functions. A slight variation in *q* yields an appreciable inverse variation in the value of *n*. It has resulted from this study that the *n* parameter decreases while the *q* increases with $\eta$.

An increasing trend is observed in the values of $N_0$ with increasing $\eta$ extracted by the fit using Tsallis function. The value varies from $93.0\pm 0.2$ to $97.1\pm 0.2$ in case of experimental data while for Pythia it yields $88.8\pm 0.2$ for $\eta = 0.1$ while $98.4\pm 0.2$ for $\eta = 2.3$. The function yield a value of $77.2\pm 0.2$ for the lower $\eta$ bin while $86.3\pm 0.2$ for the higher $\eta$ bin. The values of $N_0$ in case of the Pythia model is closer to the experimental data than the EPOS model. Since $N_0$ is proportional to the multiplicity of particles and Pythia reproduced similar multiplicity hence predicted better results than the EPOS model. This might be connected to the effects that are incorporated in the Pythia model such as flow-like effect and color reconnection effect which is produced from simultaneous hard sub-collisions forming color strings between initial and final state partons from separate hard scatterings due to which the model predicts the data well.

Before going to conclusion section we would like to clarify that the $T_0$ and $T_e{eff}$ are not different. The former does not include the flow effect, however the later includes the flow effect. We observed that both of them decrease with increasing pseudo-rapidity due to the large energy transfer in lower pseudo-rapidity intervals. The physics behind this is that larger energy transfer in the lower pseudo-rapidity bin results in higher degree of excitation of the system which results in larger $T_0$ and $T_{eff}$^10,38–41^ and this indicates that the system in lower pseudo-rapidity intervals comes to equilibrium quickly. In addition we also observed that q (n) is increasing (decreasing) with the increase of rapidity. As discussed above, q and n are reciprocal. A system with smaller q (more closer to unity) and larger *n* indicates to be more closer to equilibrium state^42–45^. In the present work the increase (decrease) of *q* (*n*) with increasing $\eta$ claims that the system goes far from the equilibrium state as the psuedo-rapidity intervals is larger. The $\beta _T$ is also observed to decrease with increasing $\eta$ due to large energy deposition in lower rapidity bins. Actually, the large deposition of energy in a system indicates its rapid expansion. In the present work, the larger $\beta _T$ in lower $\eta$ bins shows that there is large pressure gradient due to the fact that large amount of energy is transferred to the system which results in a quick expansion of the fireball.

## Summary and conclusion

Simulations of EPOS and Pythia models are performed and then compared with the measurements of the CMS experimental data in *pp* collisions at $\sqrt{s} =$ 7 TeV. The transverse-momentum ($p_T$) spectra are presented from 0.1 to 2 $\text {GeV/}c$ in twelve pseudorapidity ($\eta$) intervals from 0 to 2.4 in steps of 0.2 and the $p_T$ spectrum from 0.1 to 6 $\text {GeV/}c$ for a wider $\eta$ interval, 0 $\le$ $\eta$ $\le$ 2.4 as a single bin. The Pythia model predicts the $p_T$ spectra at all the pseudorapidity bins very well particularly at the higher value of $p_T$, while over predicts the distribution slightly at the lower $p_T$ region. The model also predicts the $p_T$ spectra for a wider range of $\eta$, $\eta$ < 2.4 and over a wide range of $p_T$, 0 $\le$ $p_T$ $\le$ 6 $\text {GeV/}c$. However, within the experimental error a slight bump is predicted in a narrow region at lower $p_T$ interval. The EPOS model under-predicts the $p_T$ spectra over the entire $p_T$ range and in all $\eta$ intervals. The model also underestimates the experimental results of $p_T$ spectra for all $\eta$ intervals for about 15% at $\eta$ < 2.4 while a reasonable agreement is shown for $p_T$ > 4.2 $\text {GeV/}c$ within the experimental errors. The Pythia model reproduce the charged particle pseudorapidity integrated over $p_T$ for the whole region of $\eta$, − 2.5 $\le$ $\eta$ $\le$ 2.5 very well whereas the EPOS model under-predicts from 10% to about 15%.

Furthermore, the models’ predictions and the experimental data are fitted by two statistical functions to get information about some collective properties of the hadronic matter. The measured experimental data and models simulations are fitted by modified Hagedorn function with embedded transverse flow velocity and thermodynamically consistent Tsallis distribution function. The two function fitted the data and models prediction very well. The values of transverse flow velocity ($\beta _T$), and Kinetic freeze–out temperature ($T_0$) extracted from the Hagedorn distribution function while effective temperature ($T_{eff}$) is obtained from the Tsallis function. The value of $\beta _T$, $T_0$, and $T_{eff}$ decrease with increasing $\eta$ because large energy transfer in the lower pseudo-rapidity bin results in higher degree of excitation of the system which results larger values of $T_0$ and/or $T_{eff}$. Furthermore, the decrease in $\beta _T$ with increasing $\eta$ is due to the large energy deposition in lower rapidity bins producing rapid expansion. In the present work, the larger $\beta _T$ in lower $\eta$ bins shows that there is large pressure gradient due to the fact that large amount of energy is transferred to the system resulting in a quick expansion of the fireball. It is concluded that the Pythia model describes the experimental data at most of the $p_T$ regions for all the $\eta$ bins, while the EPOS underpredicts mostly. The Pythia model also predicted the $p_T$ distribution over a wider $p_T$ range well which might be connected to the color re-connection and flow-like feature of the Pythia model, whereas the EPOS reproduced the distribution at higher values of the $p_T$ only and largely underpredicts the distribution. Although, the models under-study reproduced the $p_T$ distribution of the charged particles in some region of $p_T$ in different $\eta$ regions presented from 0 to 2.4 in steps of 0.2, but none of them completely describe all the distribution over the entire $p_T$ range.
